# 48218 Preclinical modeling of BRAF(V600E)/PTEN-/- melanoma leptomeningeal disease (LMD) to assess intrathecal checkpoint blockade

**DOI:** 10.1017/cts.2021.629

**Published:** 2021-03-31

**Authors:** Renato A. Guerrieri, Grant M. Fischer, Barbara G. Knighton, Courtney W. Hudgens, Debora A. Ledesma, Michael A. Davies, Sherise D. Ferguson

**Affiliations:** MD Anderson Cancer Center

## Abstract

ABSTRACT IMPACT: Melanoma leptomeningeal disease (LMD) is a devastating subtype of central nervous system (CNS) metastatic disease that is associated with limited treatment options and an extremely poor prognosis, thus requiring the development of preclinical models of LMD for therapeutic development. OBJECTIVES/GOALS:

1. Develop an immunocompetent murine model of melanoma LMD with tumors bearing genetic mutations commonly found in patients, specifically BRAF(V600E)/PTEN-/-
2. Assess the safety of intrathecal (IT) immunotherapy, specifically anti-PD1 antibody (aPD1)
3. Evaluate the therapeutic efficacy of IT aPD1 checkpoint blockade in murine melanoma LMD METHODS/STUDY POPULATION: To develop BRAF(V600E)/PTEN-/- LMD models, we acquired BP, D4M, and D4M-UV2 (irradiated) murine melanoma cell lines and luciferase-tagged them. 1.5x10^4 cells were suspended in 10 uL serum-free media and injected into the cisterna magna of female C57BL/6 mice. Brain and spinal cord were harvested for histologic assessment once mice were moribund. To assess safety of IT aPD1, we injected IT control IgG or IT aPD1 (13 ug, 26 ug, 39 ug) and monitored weights or harvested at days 7 or 14 for IHC staining of inflammation markers. To evaluate therapeutic efficacy of IT aPD1, BP cells were directly injected as above. After 3 days, mice underwent imaging to confirm tumor uptake and randomization to receive 13 ug IT control IgG or aPD1 once + 200 ug systemic (Sys) control IgG or aPD1 (days 0, 3, and 5), and then monitored for survival. RESULTS/ANTICIPATED RESULTS: For LMD development, all mice survived cisternal injection of BP, D4M, and D4M-UV2 cells and median survival was 17, 19, and 30 days, respectively. Presence of leptomeningeal deposits was confirmed for all tumor-bearing mice by IHC for MART1. For safety of IT aPD1, all mice survived the procedure and no mice displayed morbidity or >10% weight loss over 14 days of observation. IHC assessment of brain and spinal cord samples from mice treated with 13 ug aPD1 revealed focal ischemia related to injection site and no other signs of neurological damage or inflammation. IT aPD1 treatment of mice with BP leptomeningeal tumors demonstrated no significant survival advantage, although both IT aPD1 +/- Sys aPD1 had mice live up to days 29 and 26, respectively, compared to both IT control IgG +/- Sys aPD1, for which all mice died by day 22. DISCUSSION/SIGNIFICANCE OF FINDINGS: We demonstrate that cisternal injection of murine BRAF(V600E)/PTEN-/- melanoma cell lines yield LMD with reproducible survival and that treatment with IT aPD1 in this model is feasible and safe. Together these findings establish a new model to facilitate the development of more effective immunotherapy strategies for melanoma patients with LMD.

